# Host–microbiota interactions in rheumatoid arthritis

**DOI:** 10.1038/s12276-019-0283-6

**Published:** 2019-12-11

**Authors:** Yuichi Maeda, Kiyoshi Takeda

**Affiliations:** 1 Laboratory of Immune Regulation, Department of Microbiology and Immunology, Graduate School of Medicine, WPI Immunology Frontier Research Center, Osaka University, Suita, Japan; 2 Core Research for Evolutional Science and Technology, Japan Agency for Medical Research and Development, Tokyo, Japan; 3 Department of Respiratory Medicine and Clinical Immunology, Osaka University Graduate School of Medicine, WPI Immunology Frontier Research Center, Osaka University, Suita, Japan

**Keywords:** Translational research, Acute inflammatory arthritis, Rheumatoid arthritis

## Abstract

The gut microbiota has been proposed to be an important environmental factor in the development of rheumatoid arthritis (RA). Here, we review a growing body of evidence from human and animal studies that supports the hypothesis that intestinal microbiota play a role in RA. Previous studies from we and others showed an altered composition of the microbiota in early RA patients. A recent study demonstrated that *Prevotella* species are dominant in the intestine of patients in the preclinical stages of RA. In addition, *Prevotella*-dominated microbiota isolated from RA patients contributes to the development of Th17 cell-dependent arthritis in SKG mice. Moreover, it was reported that periodontal bacteria correlates with the pathogenesis of RA. In this review, we discuss the link between oral bacteria and the development of arthritis. However, many questions remain to be elucidated in terms of molecular mechanisms for the involvement of intestinal and oral microbiota in RA pathogenesis.

## Rheumatoid arthritis: gut and mouth microbes linked to autoimmune disease

Microbes living in the gut and mouth have been implicated in the development of rheumatoid arthritis (RA) and treatments that promote the growth of healthier bacterial communities may help weaken this autoimmune disease. Yuichi Maeda and Kiyoshi Takeda from Osaka University, Japan, review data from mice and humans linking RA to altered microbial compositions in the gut. They focus on a particular bacterium called *Prevotella copri*, which is found at much higher numbers in the gastrointestinal tracts of people with newly diagnosed RA than in those without the disease. Certain mouth-dwelling bacteria may also help exacerbate RA through the induction of antibodies directed against the host. The exact molecular mechanism by which gut and oral microbes contribute to RA remains unclear.

## Introduction

Rheumatoid arthritis (RA) is a systemic inflammatory disease characterized by polyarthritis that leads to joint destruction. Despite rapid progress being made in the treatment of RA^1,2^, the etiology of RA is not fully understood. It has been reported that combinations of genetic and environmental factors are involved in RA development^3,4^. The concordance rates for RA in monozygotic twins are ~15%, suggesting that environmental factors are important for RA development^5^. Exposure to many environmental factors, including smoking, hormones, microbiota, and infections, may be involved in the induction of the disease^6–11^. Among these environmental factors, the gut microbiota plays an important role in the development of arthritis in mice^12–15^. Recent studies showed that immunoglobulin A (IgA) anti-citrullinated protein (CCP) antibodies are detectable for several years before the onset of arthritis in humans^16,17^. These findings suggest that RA originates at mucosal sites, such as the gut and oral cavity. Considering that antimicrobial drugs such as minocycline or salazosulfapyridine are effective in some RA patients, the gut and oral microbiota appear to be correlated with the disease^18,19^.

Here, we review recent works showing the altered composition of the gut microbiota observed in RA patients. Moreover, we describe the correlations between the gut microbiota and human or murine arthritis in previous studies. We also discuss recent evidence that *Prevotella* species directly contribute to the development of arthritis in mice.

## Dysbiosis triggers arthritis in animal models

Several studies on experimental murine arthritis have clearly demonstrated the importance of the intestinal microbiota in the pathogenesis of arthritis (Table 1). When mice are reared in germ-free (GF) conditions or treated with antibiotics, they do not develop arthritis^12,20^. However, the inoculation of specific microbes is sufficient to induce arthritis in GF-conditioned mice^12,21,22^, suggesting that the gut microbiota plays an important role in the development of arthritis.

**Table 1:** Murine models of arthritis known to be correlated with the gut microbiota

Mice strain	Environmental condition	Mechanism of involvement of arthritis	Intestinal bacteria correlated with induction of arthritis	Ref.
SKG	GF, SPF: no arthritis conventional: arthritis	Production of auto-reactive T cells Activation of innate immunity by fungi	*Prevotella*-dominated microbiota	20, 22, 23
IL-1ra^−/−^	GF: no arthritis conventional: arthritis	Activation of TLR2 and TLR4 Th17 cells ↑ Treg cells ↓	*Lactobacillus Bifidus* *Helicobacter*	21, 24
K/BxN	GF: no arthritis SPF: arthritis	Production of GPI-antibody Th17 cell expansion in the intestine	SFB	12
CIA	ABX: reduced severity of arthritis SPF: arthritis	Production of anti-type II collagen antibody and serum inflammatory cytokines	–	27

*GF* germ-free, *SPF* specific pathogen free, *GPI* glucose-6-phosphate isomerase, *SFB* segmented filamentous bacteria, *TLR* Toll-like receptor, *Treg cells* regulatory T cells, *CIA* collagen-induced arthritis, *ABX* antibiotics, *Ref* references

Previous studies from we and others demonstrated that SKG mice, which spontaneously develop chronic T cell-mediated arthritis under conventional conditions, do not develop the disease under GF conditions^20,23^. However, a limited bacterial consortium, altered Schaedler flora, is sufficient to induce arthritis with a curdlan injection. We also showed that monocolonization of GF-SKG mice with *Prevotella copri* is sufficient to induce arthritis with a fungal injection^20^. These results indicate that a particular commensal bacterium is sufficient to induce arthritis in SKG mice.

As another model of arthritis, interleukin (IL)-1 receptor antagonist knockout (IL1rn^−/−^) mice spontaneously develop T cell-mediated arthritis under specific-pathogen-free conditions^21^. These mice do not develop arthritis under GF conditions. However, monocolonization of the mice with *Lactobacillus bifidus* induces arthritis. Recently, Rogier et al. revealed the importance of IL-1 receptor antagonists in maintaining the diversity and composition of the commensal microbiota. IL1rn^−/−^ mice display decreased bacterial richness and diversity, and their altered microbiota is characterized by a high abundance of *Helicobacter* species and a low abundance of *Ruminococcus* species. The Th17 cell population is increased in the intestinal lamina propria of IL1rn^−/−^ mice, and the phenotype is transferable to wild-type mice. Tobramycin treatment decreases the abundance of the commensal microbiota, such as *Helicobacter* species, and suppresses arthritis in IL1rn^−/−^ mice. Furthermore, by using IL1-rn and TLR4 double-knockout mice, the dysbiosis in IL1rn^−/−^ mice was shown to be TLR4-dependent^24^.

K/BxN T cell receptor transgenic mice develop inflammatory arthritis with high titers of autoantibodies directed against glucose-6-phosphate isomerase^25,26^. When the mice are reared under GF conditions, they do not develop the disease and display reduced numbers of Th17 cells in the small intestine and spleen^12^. Monocolonization with segmented filamentous bacteria is sufficient to cause Th17 cell-dependent arthritis in these mice.

Recently, Widian et al. reported that intestinal dysbiosis triggers collagen-induced arthritis (CIA) via mucosal immune responses. Dysbiosis and mucosal inflammation precede the development of CIA^27^. Treatment with antibiotics was found to reduce the disease severity, as well as the levels of anti-type II collagen antibodies and serum inflammatory cytokines. Therefore, certain gut commensal microbiota is sufficient to induce arthritis in mice. However, more intensive analyses are needed to analyze which bacterium shows a strong effect on the development of arthritis.

## Dysbiosis in human RA patients

**Fig. 1:**
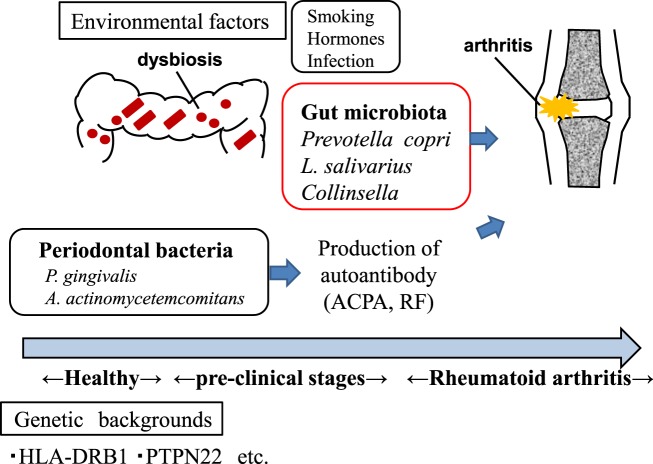
Both genetic and environmental factors are involved in the pathogenesis of arthritis. The gut and oral microbiota may contribute to the development of arthritis. *P. gingivalis* *Porphyromonas gingivalis*, *A. actinomycetemcomitans* *Aggregatibacter actinomycetemcomitans*, *L. salivarius* *Lactobacillus salivarius*, ACPA anti-citrullinated protein antibodies, RF rheumatoid factor

Recent accumulating evidence supports the hypothesis that the gut microbiota plays a pivotal role in the development of human arthritis (Fig. 1). Several case–control studies have shown that the composition of the intestinal microbiota is altered in RA patients (Table 2).

**Table 2:** Altered composition of the gut microbiota in human RA patients

Country	Increased bacteria	Reduced bacteria	Method	Ref.
USA	*Prevotella (Prevotella copri)*	*Bacteroides*	16S rRNA sequencing	30
Japan	*Prevotella (Prevotella copri)*	*Bacteroides*	16S rRNA sequencing	20
USA	*Collinsella*	*Faecalibacterium*	16S rRNA sequencing	34
China	*Lactobacillus salivarius* etc.	*Veillonella, Haemophilus* etc.	Metagenomic shotgun sequence	32

Vaahtovuo et al.^28^ analyzed the composition of the microbiota in patients with untreated early RA or fibromyalgia using a technique based on flow cytometry, 16S rRNA hybridization, and DNA staining. In the *Bacteroides fragilis* subgroup, the genera *Bifidobacterium* and *Eubacterium rectale*–*Clostridium coccoides* were decreased in RA patients. These results are comparable to previous results in patients with Crohn’s disease^29^.

Scher et al.^30^ found using 16S rRNA gene sequencing that patients with untreated new-onset RA in American populations harbored an increased abundance of *P. copri* and a reduced abundance of *Bacteroides* species in the intestine. Interestingly, the relative abundance of *P. copri* was inversely correlated with the presence of shared epitope risk alleles. We further found that some Japanese patients with recent-onset RA carry an increased abundance of the genus *Prevotella*, especially *P. copri*, and a decreased abundance of *Bacteroides* species in the intestine^20^. Very recently, preclinical phase RA patients in European countries were shown to harbor a high abundance of *Prevotella* species, including *P. copri*, in the intestine, suggesting that dysbiosis precedes the development of arthritis^31^.

A study in China demonstrated that RA patients had an increased abundance of *Lactobacillus salivarius* in the gut, on the teeth, and in the saliva, based on metagenomic shotgun sequencing^32^. In contrast, *Haemophilus* species were found to be depleted at all three sites in RA patients. The abundance of *P. copri* in the gut was elevated in the first year after disease onset. Interestingly, the dysbiosis observed in RA patients was partially restored after treatment with disease-modifying drugs. Furthermore, in China, Liu et al.^33^ found that fecal *Lactobacillus* species were enriched in RA patients compared with healthy controls (HCs).

Chen et al. reported that compared with HCs, patients with RA show decreased gut microbial diversity, which correlates with autoantibody levels and disease duration. Interestingly, methotrexate induces an increase in species richness and diversity. The relative abundance of *Collinsella* was found to be increased in RA patients. In contrast, *Faecalibacterium*, which is generally recognized as a beneficial microbe, is decreased in RA patients. Inoculation of *Collinsella* into CIA-susceptible mice induces severe arthritis. In vitro experiments showed that *Collinsella* increases gut permeability and induces IL-17A expression, suggesting that *Collinsella* is a candidate arthritogenic bacterium in the human intestine^34^. In summary, *P. copri, L. salivarius, and Collinsella* are the dominant gut microbiota in patients with early RA and may be involved in its pathogenesis. The reason for the different candidate arthritogenic intestinal bacteria is possibly due to the host genetic background and environmental exposures, such as diet.

## *Prevotella copri*: a possible trigger of RA

Several reports have described the altered composition of the microbiota in RA patients. Among these studies, we and others found that *Prevotella* species, especially *P. copri*, are the dominant fecal microbiota in early RA patients^20,30^. Scher et al.^30^ have shown that *P. copri* exacerbates murine colitis in mice administered dextran sulfate sodium in their drinking water. However, it remains unclear whether the dysbiosis observed in RA patients triggers the development of arthritis.

To answer this question, a novel approach was taken to generate the intestinal microbiota: humanized mice^20^. Fecal samples were anaerobically obtained from early RA patients and HCs, diluted, and orally inoculated into GF-SKG mice. SKG mice develop T cell-mediated arthritis by the activation of innate immunity, resembling human RA^23^. Both *Prevotella*-dominated RA microbiota and HC microbiota successfully colonized GF-SKG mice. The SKG mice colonized with a *Prevotella*-dominated microbiota from RA patients (RA-SKG mice) showed severe arthritis and increased numbers of Th17 cells in the large intestine. Lymphocytes isolated from popliteal lymph nodes and the large intestine secreted IL-17 in response to the arthritis-related autoantigen RPL23A. In vitro analyses revealed that *P. copri* had the ability to induce the production of Th17-related cytokines such as IL-6 and IL-23^20^. Thus, these data strongly indicate that *Prevotella* species, especially *P. copri*, trigger the development of arthritis. Further analyses are needed to investigate whether intestinal barrier or immune cell populations are altered in patients during the initial stages of RA.

Recently, using liquid chromatography–tandem mass spectrometry, Pianta et al.^35^ identified a novel HLA-DR-presented peptide in a 27-kDa *P. copri* protein (Pc-p27) from peripheral blood mononuclear cells of RA patients. Pc-p27 stimulated Th1 responses in 42% of RA patients, although the authors did not show correlations between these proteins and *P. copri* abundance in the intestine. A subgroup of RA patients showed IgA responses to Pc-p27 or whole *P. copri* cells. Interestingly, a subgroup of RA patients had *P. copri* 16S rDNA in their synovial fluid. The authors further identified two novel HLA-DR-presented peptide autoantigens, N-acetylglucosamine-6-sulfatase (GNS) and filamin A (FLNA)^36^. T cell and B cell responses to GNS and FLNA were observed in 52% and 56% of RA patients, respectively. Interestingly, the GNS and FLNA HLA-DR-presented T cell epitopes have sequence homology with *Prevotella* epitopes. Moreover, GNS and FLNA autoantibodies positively correlated with *P. copri* antibody levels. Thus, the authors clearly demonstrated a relationship between microbial peptides from gut commensal bacteria and autoimmune responses affecting joints.

By contrast, several studies have demonstrated that the genus *Prevotella* is one of the major commensal bacteria in healthy subjects and plays beneficial roles in the host. In Africa and tropical Asia, healthy individuals were found to harbor a high abundance of *Prevotella* in the intestine^37^. It has been reported that the human gut microbiota can be divided into three enterotypes^38^, characterized by high levels of *Bacteroides, Prevotella*, and *Ruminococcus*. Therefore, *Prevotella* species are detectable in the intestine of RA patients and also some healthy individuals. It would be an interesting future issue to clarify which *Prevotella* species component leads to the development of arthritis.

A recent study demonstrated that *Prevotella histicola* from the intestine of healthy humans decreased the severity of CIA in HLA-DQ8 mice^39^. HLA-DQ8 mice were immunized with collagen and orally inoculated with *P. histicola*. The mice treated with *P. histicola* showed ameliorated arthritis through a reduction in intestinal permeability. *P. histicola* increased regulatory T cell numbers in the gut and reduced antigen-specific Th17 responses. The sequences of *P. histicola* were completely different from those of *P. copri*. These results indicate that some *Prevotella* species, such as *P. histicola*, can suppress the induction of arthritis. In summary, *P. copri* and *P. histicola* show different effects on arthritis. These differences might derive from the genetic diversity among *Prevotella* species.

## Correlation between periodontal bacteria and arthritis

Recent studies have revealed that periodontal disease is correlated with an increased risk of RA in humans and in mouse models of arthritis^40–42^. The presence of periodontitis in patients with RA is associated with anti-CCP antibody levels^43^. Moreover, periodontitis is correlated with the disease activity of RA^44^. In addition, treatment of periodontitis ameliorates the disease activity of RA^45,46^. These results suggest that periodontal bacteria are correlated with RA pathogenesis.

*Porphyromonas gingivalis*, one of the major periodontal bacteria, is the only known pathogen that expresses a bacterial peptidylarginine deiminase^47–49^. Reports have shown that *P. gingivalis* infection positively correlates with the production of anti-CCP antibody responses in RA patients^50,51^. In a CIA model, oral inoculation of *P. gingivalis* was found to exacerbate arthritis through the increased production of IL-17^42^. The arthritogenic effects of *P. gingivalis* are dependent on the bacterial strain, presence of fimbriae, and time of infection^52^. Recently, Sato et al.^53^ showed that *P. gingivalis*, but not *Prevotella intermedia*, exacerbate arthritis by modulating the gut microbiota and increasing the proportion of Th17 cells in mesenteric lymph nodes. However, the roles of other pathogens in the development of arthritis have not been fully investigated.

*Aggregatibacter actinomycetemcomitans*, a periodontal bacterium, was recently proposed to connect periodontitis to RA because of its ability to induce citrullinated autoantigens. Konig et al. reported that the pore-forming toxin leukotoxin-A produced by *A. actinomycetemcomitans*, but not by other periodontal pathogens, drives hypercitrullination in neutrophils^54^. Moreover, antibodies against *A. actinomycetemcomitans* and leukotoxin-A were found to be highly detectable in human RA patients. In summary, periodontal bacteria such as *P. gingivalis* and *A. actinomycetemcomitans* may contribute to autoantibody production and autoimmunity in RA. Further analyses are needed to elucidate whether A. *actinomycetemcomitans* induces anti-CCP antibody production in vivo.

## Conclusion

In this review, we have summarized the role of the intestinal microbiota in human RA and in murine models of arthritis. Several studies have demonstrated that *P. copri* is present in early RA patients and contributes to the induction of arthritis. However, the precise molecular mechanisms by which *P. copri* exacerbates human arthritis are still unknown. *L. salivarius* and *Collinsella* were found to be the dominant gut microbiota in other cohorts. Moreover, it was reported that periodontal bacteria such as *P. gingivalis* and *A. actinomycetemcomitans* may induce the production of anti-CCP antibodies, leading to the development of arthritis. Further studies are needed to clarify the mechanistic links between these specific bacteria and RA development in humans. The manipulation of dysbiosis would be a novel preventative strategy in RA patients.
